# Incidence of malarial infection and response to antimalarial drugs at Districts Lower Dir and Swat of Khyber Pakhtunkhwa, Pakistan

**DOI:** 10.1016/j.dialog.2022.100035

**Published:** 2022-08-12

**Authors:** Nasib Zaman, Faiz Ul Haq, Zafran Khan, Wajahat Uallah, Daniya Ualiyeva, Yasir Waheed, Muhammad Rizwan, Raja Tahir Mahmood, Majid Mahmood

**Affiliations:** a Center for Biotechnology and Microbiology, University of Swat, Pakistan; b University of Chinese Academy of Sciences, Beijing 100049, China; c State Key Laboratory of Respiratory Disease, Guangzhou Institutes of Biomedicine and Health, Chinese Academy of Sciences, Guangzhou 510530, China; d Chengdu Institute of Biology, Chinese Academy of Sciences, Chengdu 610041, China; e Foundation University Medical College, Foundation University Islamabad, Islamabad, Pakistan; f Department of Biotechnology, Mirpur University of Science and Technology (MUST), Mirpur 10250 (A.J.K.), Pakistan; g Department of Zoology, The University of Poonch, Rawalakot, Azad Jammu and Kashmir 12350, Pakistan

**Keywords:** Malaria, Incidence, *Plasmodium falciparum*, *Plasmodium vivax*, Antimalarial drugs, Chloroquine

## Abstract

Malaria is the leading cause of mortality and morbidity all over the world. Several antimalarial drugs are available for the treatment of malaria. The main objective of this study was to investigate the incidence of malarial infection and the use of prescribed antimalarial drugs. A cross-sectional study was carried out to collect quantitative data from selected sites in District Lower Dir and Swat of Malakand Division Khyber Pakhtunkhwa (K.P.), Pakistan. Screening of selected patients was performed using both thick and thin films and was observed with the help of a light microscope. In this study, a total of 2517 blood samples were tested. Overall positive infection was 12% *Plasmodium vivax* (99.07%) and *Plasmodium falciparum* (0.92%). Our results evaluate that infection with *Plasmodium vivax* was higher than *Plasmodium falciparum.* No other Plasmodium species or mixed infections were observed. The rate of infection was more frequent in males as compared to female patients. The highest percentage was recorded in the summer season (35.07%), while the lowest was documented in the winter (11.7%). Out of 325 patients, 311 (95.7%) were treated with Chloroquine, and the remaining were treated with Artemether. Chloroquine was used as a drug of choice for *Plasmodium vivax* infection. The present study concludes that *Plasmodium vivax* and *Plasmodium falciparum* are the two common agents for malaria in Malakand Division. However, *Plasmodium vivax* was dominant over *Plasmodium falciparum*. The infection rate was high in males from District Lower Dir during the summer season.

## 1 Introduction

Malaria is one of the deadliest infectious diseases, with more than 200 million clinical cases each year and 600,000 death in children and women in the World [1]. According to the latest World malaria report, released in December 2019, there were 228 million malaria cases in 2018 compared to 231 million cases in 2017. The estimated number of malaria deaths stood at 405000 in 2018, compared with 416,000 deaths in 2017 [2]. The world is currently not on track to achieve the milestones of 2020 of World Health Organization (WHO) Global Technical Strategy (G.T.S.) of malaria 2016–2030. The target of 2020 is to reduce malaria death and disease by 40%, but there is a continuous increase in malaria cases from 2015 to 2018. The number of malaria cases globally increases by 214, 217, and 219 million from 2015, 2016, and 2017 [1].

Pakistan has a population of 180 million inhabitants, of which 177 million are at risk of malaria. With 3.5 million presumed and confirmed malaria cases annually, Pakistan is included in the endemic list of countries for malaria. In Pakistan, *Plasmodium vivax* is more common, followed by *Plasmodium falciparum* which is reported mostly in Baluchistan, southern Punjab, and Sindh province [3]. Major reports of malarial infection are from the border area of Pakistan with Afghanistan and Iran, in which *P.vivax and P.falciparum* are the most prevalent species [4].

Malarial infection in humans results from five known species of the Plasmodium genus, which are *Plasmodium falciparum (Pf), Plasmodium Vivax (Pv), Plasmodium Ovale (Po), Plasmodium malariae(Pm), and Plasmodium knowlesi (Pk)* [5]. *Plasmodium knowlesi* is a zoonotic simian species that occasionally infect humans [6,7]. Female mosquito *Anopheles* responsible for its transmission [8]. Among all anopheles' species, 41 are the most important malarial vector which is to be considered dominant vector species (D.V.S.) [9]. Malaria is a potentially life-threatening disease, especially in pregnant women and children under five years, the most affected group [3].

Severe malarial infection results in acute respiratory failure, hypoglycemia, pulmonary edema, renal failure, severe anemia, unarousable coma, seizures, and jaundice [10,11].

Quinine was the first antimalarial drug which is isolated from the cinchona tree [[12], [13]]. Chloroquine has been the drug of choice in all uncomplicated malaria cases for more than fifty years [14]. It is reported from Pakistan that Chloroquine is active against *P. vivax*, but some resistance is also reported [15]. Artemisinin is used as a blood schizonticide that prevents the development of pathological affliction for acting on early young stages and containing activity in the erythrocyte stage [16].

This study aimed to determine the response and resistance of prescribed antimalarial drugs and to assess the incidence and infected population at Malakand Division. The study was also conducted to investigate seasonal/monthly variation of malarial infection.

## 2 Materials & methods

### 2.1 Study area

The study was carried out in two districts, i.e., Swat and Lower Dir of Malakand Division Khyber Pakhtunkhwa. The study area was selected based on the availability of data and distance from the University of Swat (U.O.S.). District Swat is located at a latitude of 35.2227 N and longitude 72.4258E. District Dir Lower is located at a latitude of 34.9161 N and longitude 71.8097E.

### 2.2 Study design and data collection

This cross-sectional study was conducted from December 2017 to November 2018. Informed written consent was taken from all the patients to participate in the study. Data were collected from District Health Officer Malaria laboratory Saidu Sharif, Saidu Central Hospital, D.H.Q. Hospital Timergara and other health care centers of both districts. These are the government sector related to dengue and malaria control, most of the patients with malaria are admitted here. The study was approved by the Ethical Committee of Centre for Biotechnology & Microbiology (C.B.M.), University of Swat, Pakistan.

### 2.3 Selection criteria

**Fig. 1:**
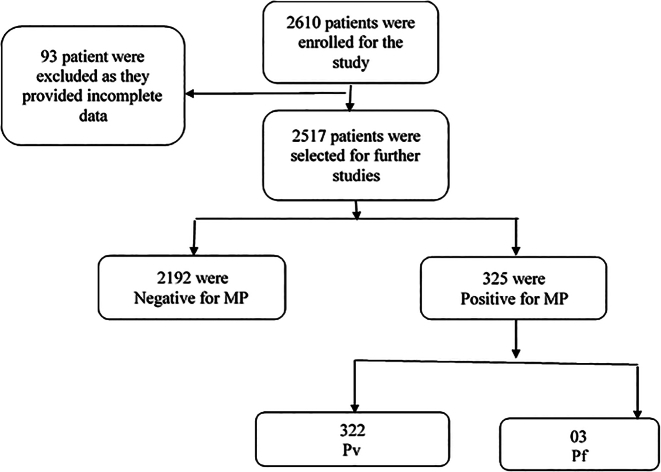
Flow sheet diagram 2517 patients who have received treatment. Note: MP = Malarial Parasites, Pv = *Plasmodium vivax*, Pf = *Plasmodium falciparum.*

The study included participants of all age groups, male and female, with malarial signs and symptoms (fever, headache, chills, nausea, vomiting, anemia, sweats, and fatigue). Patients from other areas of the Malakand Division were excluded from our study. The flow chart for the collection is shown in Fig. 1.

### 2.4 Clinical assessment and questionnaire survey

Detailed information was collected from all patients by filling in written questionnaires distributed before sample collection. The questionnaire contains information related to the patient and the drugs used. The data about sociodemographic profile, clinical history about symptom, previous malaria infecyion, reinfection and drugs were noted. The patients were rechecked after two to three weeks, and history was recorded about the drugs used and the effect of drugs against malaria.

### 2.5 Sample collection

About 3 ml of whole intravenous blood was drawn using a new and sterile syringe from each enrolled participant. Blood was immediately added to the EDTA tube and properly labeled. The samples were transferred to the C.B.M. lab for further processing.

### 2.6 Smears preparation and staining

One to two drops of blood were placed on slides, and then by using another slide (spreader), spread the blood, and thin smears were made and allowed to air dry. The films were fixed in ethanol. Thick films were made by spreading the blood with a match stick and air-drying. The thick films were not fixed in alcohol. Giemsa staining (10%) was performed in order to stain both films. Then cells were observed to identify Plasmodium parasites under a microscope using 100 X (Oil immersion) objective.

### 2.7 Data analysis

Data generated from the questionnaire and laboratory investigations were statistically analyzed using Statistical Package for Social Sciences (SPSS) software version 18 and the M.S. Excel program. Descriptive statistic were used for frequency and percentage.

## 3 Results

### 3.1 The overall incidence of malarial infection at two sites of Malakand Division

In this study, we screened 2517 patients for the presence of malaria parasite, out of which 325 (12.91%) patients showed positive results and 2192 (87.1%) showed negative results for the presence of malaria. *Plasmodium vivax* was found in 322 (99.07%) patients, and *Plasmodium falciparum* was found only in 03 (0.93%) patients, as shown in Table 1. In this study, positive malarial cases were observed from both districts of the Malakand Division. However, the rate of positive malaria cases was higher in patients from district Lower Dir than district Swat ((19.7% vs.9.11%).

**Table 1:** District wise incidence of malaria parasite infections in study area (District Swat, District Lower Dir).

District	Total suspected N (%)	Infected N (%)	P.*vivax* N (%)	P.*falciparum* N (%)
Swat	1613 (64)	147 (9.11)	145 (98.63)	2 (1.36)
Dir Lower	904 (36)	178 (19.7)	177 (99.43)	1 (0.57)
Total	2517	325 (12.91)	322 (99.07)	3 (0.92)

### 3.2 Gender and season wise incidence of malaria

A total of 2517 patients were enrolled in this study, out of which 1614 (64.1%) were male, and 903 (35.9%) were female. High rate of positive malaria was found in male (13.3%) than in female (12.3%) patients, as shown in Fig. 1. Among 325 positive samples, the positivity rate was high in the summer season 114 (35.07%), followed by Autumn 113 (34.76%), and low infection was found in winter season 38 (11.7%), as shown in Table 2.

**Table 2:** Seasonal wise variation of malarial infection at two districts.

District	District Swat		District Lower Dir	
Seasons	Total sample	Positive	Total sample	Positive
Summer	563	86 (15.27%)	254	28 (11.02%)
Autumn	06	02 (33%)	342	111 (32.45%)
Winter	303	17 (5.61)	146	21 (14.38%)
Spring	741	42 (5.66%)	162	18 (11.11%)
Total	1613	147 (9.11%)	904	178 (19.69%)

### 3.3 Efficacy and duration of drugs against malarial parasites

In this study, patients were treated with different antimalarial drugs. Positive treatment outcome was achieved in 320 (98.5%) patients, and resistance was observed in 5 (1.5%) patients. Rate of treatment effectiveness in male were 210 (98%) and resistance 4(2%) against malarial parasites while in female the treatment effectiveness in were 110(99%) and resistance 1(0.9%). Out of 325 positive malarial patients, 311 were treated with Chloroquine, and the remaining 14 patients were treated with Artesunate and Artemether and nivaquine syrup. Among 14 patients, 06 patients were treated with Artesunate and Artemethe and the 08 patients were treated with nivaquine syrup. Among 325 patients 05 patients develop resistance to the drug, in which 04 (1.28%) develop resistance to chloroquine out of 311 patients, while there was no resistance to Artesunate and Artemether and only 01 patients out of 08 develop resistance to nivaquine syrup. The treatment response of chloroquine in malaria patients were good after 72 h (45.98%) of taking medication, however 31.51% patients show good response to chloroquine after 48 h of treatment, and the lowest rate was observed in 24 h. The patient showed resistance to Chloroquine and was retreated with other alternative antimalarial drugs. Nivaquine syrup was recommended to some children, as shown in Table 3.

**Table 3:** Effectiveness of Chloroquine, Artesunate, Artemether and Nivaquine drugs against malarial parasites in different duration of treatment.

Used Drug	24 h	48 h	72 h and above	Resistance	Total
Chloroquine	66(21.22%)	98(31.51%)	143(45.98%)	04(1.28%)	311
Artesunate and Artemether	01(16.66%)	02(33.33%)	03(50%)	–	06
Nivaquine syrup	01(12.5%)	02(25%)	04(50%)	01(12.5%)	08

### 3.4 Observation of Plasmodium falciparum under a microscope

**Fig. 2:**
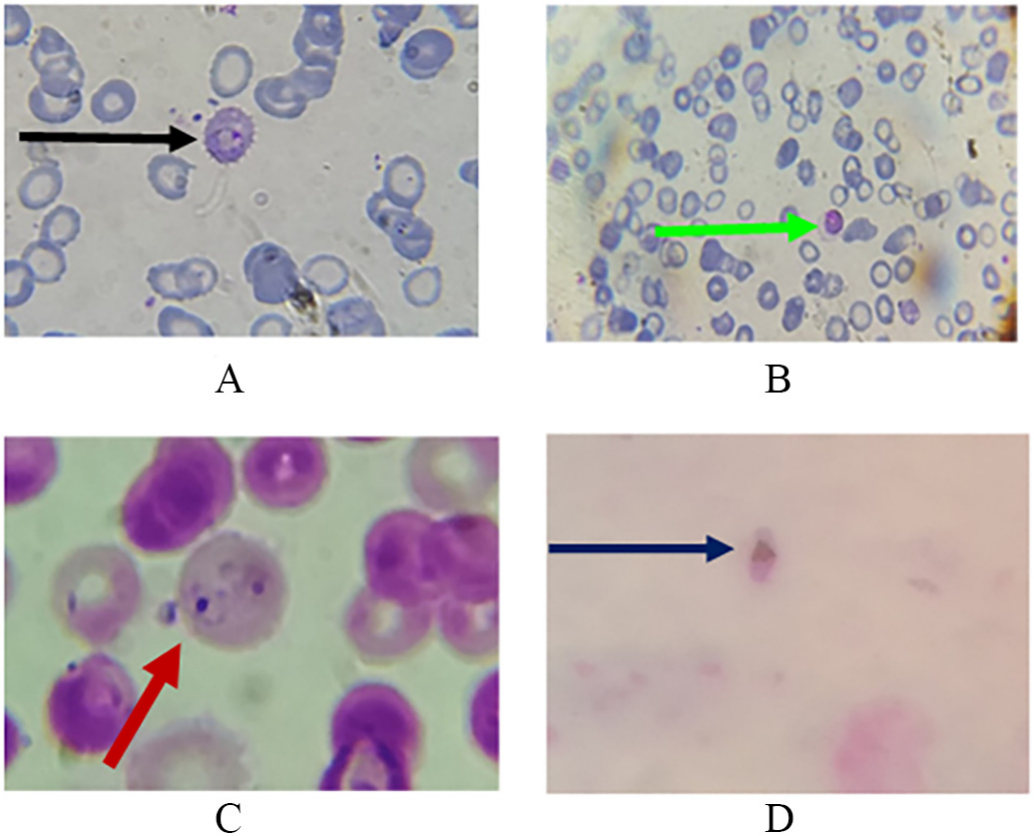
Identification of Plasmodium *falciparum* under microscope.

*Plasmodium falciparum* was observed in different stages under a microscope, including Tropozoite form, Shizonts, and Gametocyte stage. The different forms of *Plasmodium falciparum* are shown in Fig. 2A-D.

### 3.5 Observation of Plasmodium vivax under a microscope

**Fig. 3:**
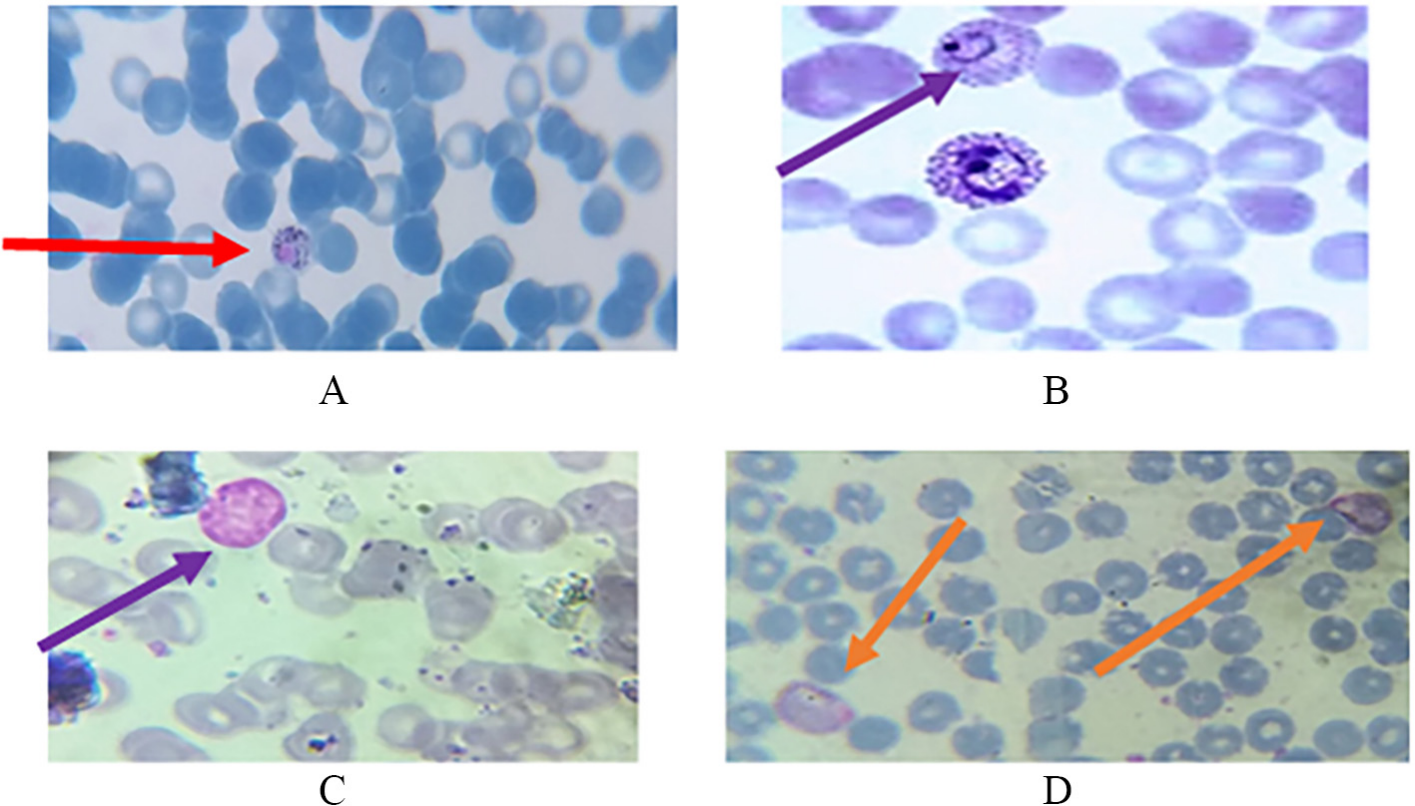
Identification of Plasmodium *vivax* under microscope.

*Plasmodium vivax* was observed in their different stages. Schizont and trophozoites of *Plasmodium vivax* were seen in thin blood smear as shown in Fig. 3A and B, respectively. Schizont and gametocyte stages of *Plasmodium vivax* were also observed and shown in Fig. 3C and D, respectively.

## 4 Discussion

Malaria is one of the most serious and major health problems in some countries like Pakistan. It is estimated that more than half a million cases are reported annually from malaria, which causes fifty thousand deaths annually [17]. It is difficult to estimate the exact number of cases in the Pakistani population due to the diversity of species in this region. This study was performed to know the status of malaria prevalence in Pakistan. In this study, we investigated the 12.91% rate of malaria in Swat and Lower Dir of K.P., which is closely related to the investigation reported by Shah and his group. However, the rate is very high than the rate reported in Swat (19.7%) [18]. The rate of incidence in our study is in agreement with the incidence previously reported by Ahmad (17.32%) [19], (39.5%) [18]. and [24]. However, the rate is higher than the rate (9%) in the same region reported [20].

We observed a consistent rate of infection in Lower Dir and an increase in malaria in district Swat. In our study, we found two species of Plasmodium, which are P. f*alciparum* and *P. vivax*. These findings are similar to the finding of other studies conducted previously in Pakistan [21,22]. In our current study, *P. vivax* (99.07%) was predominantly observed compared to *P. falciparum* 0.93%*.* Our study is in accordance with the study of Shah and his colleagues [18,23]. However, in their study, the infection rate of *P. falciparum* is high than our study (3.9%). The incident rate in our study is different from the study of Khan and his colleagues, as they did not report a single case of *P. falciparum* [20].

*Plasmodium vivax* is more common at Malakand division because the selected study region was a temperate and subtropic region, so *Plasmodium vivax* is mostly adopted in the temperate and subtropical region while *P. falciparum* is tropical species that is found rear and does not exceed too much to temperate region. In this study, we could not find a single case of mixed infection of *P. vivax and P. falciparum*, which contradicts previous studies [[24], [25], [26]]. Another study is in agreement with our study [18].

Our study reported that the antimalarial (Chloroquine) drug was 98.71% effective against *P. vivax.* Similar findings were also investigated by Bahadar and his group in K.P [27]. However, the effectiveness of Chloroquine in our study is slightly high (92.78%). [15] study also suggests that *vivax* is sensitive to Chloroquine [15]. The possible reason for Chloroquine resistance may be due to inappropriate use of drugs and the use of a drug that is not according to recommended protocol due to lack of knowledge about malaria. In our study, most of the patient was treated with Chloroquine (95.69%). The reason is that Chloroquine is cost-effective as compared to other drugs, and our society has mostly comprised of poor people.

According to our current research work, the rate of malarial infection was slightly high in male patients, 114 (13.3%) as compared to 111 (12.3%) females. Similar results were also reported in a previous study [23], which also showed that males (13.3%) are more infected than females (10.7%); Qureshi also reported a high rate of malaria in males patients than females [28]. The possible reason for this may be due to the activities performed by males, such as working outside homes, working in mines or fields, and more chances of being bitten by the mosquitoes. Also, the males are exposure to Anopheles bites and are not so covered as compared to females. Other researchers also reported such reports from District Lower Dir and other areas of Khyber Pakhtunkhwa [18,19,29,30].

The current data reveal that malaria infections are highest (32.47%) in the autumn season (September–November) and less (11.7%) in the winter season. Another study [31] also presents the same results, (39.90%) summer season and (9%) in the winter season. The reason is maybe due to rain, which suddenly increases malarial infection in the rainy season. Low temperature and dry season may be the reason for the lowest infection rate in the winter season, which slows down sporogony development [20,32].

The current study suggests that the infecting rate of malaria is high (19.7%) at district Lower Dir from district Swat Khyber Pakhtunkhwa, Pakistan (9.11%). It is maybe because district Dir lower share border with Afghanistan on its West. There is a camp of Afghan refugees at Lower Dir and who migrate from Afghan and bring a high load of malarial infection [33] also concluded that Afghan refugees have a high risk of infection from malaria because they bring infection during migration from Afghanistan. The second reason may be a lack of public awareness and inadequate healthcare facilities in the study area.

## 5 Conclusions

The present study concludes that *Plasmodium vivax* and *Plasmodium falciparum* are familiar species for malarial infection at Malakand Division. *Plasmodium vivax* was dominant over *Plasmodium falciparum* in these areas. *Plasmodium vivax* is getting resistant to Chloroquine in the selected area, but still, Chloroquine is the drug of choice to treat malaria caused by *Plasmodium vivax*. The infection rate was high in males from district lower Dir during the summer season.

## Ethical approval

The study was conducted after obtaining ethical approval from the Centre for Biotechnology and Microbiology, University of Swat, Pakistan.

## Funding

None.

## Declaration of Competing Interest

The authors declare that they have no competing interests.
